# The bovine leukemia virus microRNAs permit escape from innate immune response and contribute to viral replication in the natural host

**DOI:** 10.1186/1742-4690-12-S1-O9

**Published:** 2015-08-28

**Authors:** Nicolas A Gillet, Malik Hamaidia, Alix de Brogniez, Gerónimo Gutiérrez, Nathalie Renotte, Michal Reichert, Karina Trono, Luc Willems

**Affiliations:** 1 Molecular and Cellular Epigenetics, Interdisciplinary Cluster for Applied Genoproteomics (GIGA) of University of Liège (ULg), B34, 1 avenue de l’Hôpital, 4000 Sart-Tilman Liège, Belgium; 2 Molecular and Cellular Biology, Gembloux Agro-Bio Tech, University of Liège (ULg), 13 avenue Maréchal Juin, 5030 Gembloux, Belgium; 3 Instituto de Virología, Centro de Investigaciones en Ciencias Veterinarias y Agronómicas, INTA, C.C. 1712 Castelar, Argentina; 4 National Veterinary Research Institute (PIWet), 57 Partyzantów Avenue, 24-100 Pulawy, Poland

## 

In the natural host (Bos taurus), infection with bovine leukemia virus (BLV) remains mostly asymptomatic, resulting in a benign lymphocytosis only in about one-third of infected animals and even less frequently in a B-cell leukemia/lymphoma (10% of cases). BLV can also be experimentally transmitted to sheep that almost invariably develop leukemia/lymphoma after shorter latency periods. Upon integration, the BLV provirus becomes transcriptionally silent except for the microRNAs that remain very abundantly expressed. We used reverse genetics to evaluate the role of the viral microRNAs in the natural and experimental hosts. A BLV lacking the microRNAs replicated at wild-type levels in sheep, indicating that these sequences were dispensable. Surprisingly, the microRNAs were required for efficient replication in cows, thereby underlining the importance of studying viral determinants in the natural host. To understand the mechanisms associated with the microRNAs, we performed high-throughput RNA-sequencing of transgenic B cell lines and peripheral blood mononuclear cells isolated from cows infected either by wild-type or by isogenic microRNA-deleted viruses. Bioinformatic analyses revealed that BLV microRNAs modulate a series of pathways that include B-cell signaling and immunity. Reporter assays showed that the microRNAs target granzyme A and c-FOS transcripts and downregulate indirectly Annexin A1 and phosphoinositide-3-kinase PIK3CG. Finally, expression of the microRNAs in B-lymphocytes was associated with a decrease in phagocytosis by primary bovine macrophages. These studies thus assign a functional relevance of the BLV microRNAs in viral evasion from the innate immune response in its natural host.

